# Calcium dysregulation, functional calpainopathy, and endoplasmic reticulum stress in sporadic inclusion body myositis

**DOI:** 10.1186/s40478-017-0427-7

**Published:** 2017-03-22

**Authors:** David R. Amici, Iago Pinal-Fernandez, Davi A. G. Mázala, Thomas E. Lloyd, Andrea M. Corse, Lisa Christopher-Stine, Andrew L. Mammen, Eva R. Chin

**Affiliations:** 1 Department of Kinesiology, University of Maryland College Park, College Park, MD USA; 2 Muscle Disease Unit, National Institute of Arthritis and Musculoskeletal and Skin Diseases, Bethesda, MD USA; 3 Children’s National Health System, Children’s Research Institute, Center for Genetic Medicine Research, Washington DC, USA; 4 Department of Neurology, Johns Hopkins University School of Medicine, Baltimore, MD USA; 5 Department of Medicine, Johns Hopkins University School of Medicine, Baltimore, MD USA

**Keywords:** Myositis, Inclusion body, Muscular diseases, Calcium, Calpain, Unfolded protein response

## Abstract

Sporadic inclusion body myositis (IBM) is the most common primary myopathy in the elderly, but its pathoetiology is still unclear. Perturbed myocellular calcium (Ca^2+^) homeostasis can exacerbate many of the factors proposed to mediate muscle degeneration in IBM, such as mitochondrial dysfunction, protein aggregation, and endoplasmic reticulum stress. Ca^2+^ dysregulation may plausibly be initiated in IBM by immune-mediated membrane damage and/or abnormally accumulating proteins, but no studies to date have investigated Ca^2+^ regulation in IBM patients. We first investigated protein expression via immunoblot in muscle biopsies from IBM, dermatomyositis, and non-myositis control patients, identifying several differentially expressed Ca^2+^-regulatory proteins in IBM. Next, we investigated the Ca^2+^-signaling transcriptome by RNA-seq, finding 54 of 183 (29.5%) genes from an unbiased list differentially expressed in IBM vs. controls. Using an established statistical approach to relate genes with causal transcription networks, Ca^2+^ abundance was considered a significant upstream regulator of observed whole-transcriptome changes. Post-hoc analyses of Ca^2+^-regulatory mRNA and protein data indicated a lower protein to transcript ratio in IBM vs. controls, which we hypothesized may relate to increased Ca^2+^-dependent proteolysis and decreased protein translation. Supporting this hypothesis, we observed robust (4-fold) elevation in the autolytic activation of a Ca^2+^-activated protease, calpain-1, as well as increased signaling for translational attenuation (eIF2α phosphorylation) downstream of the unfolded protein response. Finally, in IBM samples we observed mRNA and protein under-expression of calpain-3, the skeletal muscle-specific calpain, which broadly supports proper Ca^2+^ homeostasis. Together, these data provide novel insight into mechanisms by which intracellular Ca^2+^ regulation is perturbed in IBM and offer evidence of pathological downstream effects.

**Electronic supplementary material:**

The online version of this article (doi:10.1186/s40478-017-0427-7) contains supplementary material, which is available to authorized users.

## Introduction

Sporadic inclusion body myositis (IBM) is the most common acquired myopathy in the elderly [22]. Phenotypic characteristics of IBM patients include both proximal and distal weakness and atrophy, typically worst in the knee extensors and finger flexors [35]. Histologically, IBM biopsies often display cytoplasmic protein “inclusions,” rimmed vacuoles, mitochondrial abnormalities, increased MHC type I expression, and infiltrating CD8^+^ T cells [22]. The molecular pathogenesis of IBM remains unclear and is controversial, but likely involves both inflammatory and myodegenerative processes [5, 14]. However, unlike in other inflammatory myopathies (i.e. dermatomyositis (DM), polymyositis, and immune-mediated necrotizing myopathies), immunosuppressive therapy is not beneficial in the vast majority of IBM cases, suggesting that disease progression occurs independent of the inflammatory response [5]. Currently, there are no widely-accepted animal models of IBM that recapitulate human disease. A better mechanistic understanding of myodegeneration in IBM may allow development of relevant animal models and identification of new avenues for therapy.

In healthy skeletal muscle fibers, low resting intracellular calcium (Ca^2+^) concentrations (~50nM) are tightly maintained by an array of Ca^2+^ sensors, channels, transporters, and buffers [9]. When this regulation is perturbed, prolonged cytosolic Ca^2+^ elevations can cause various downstream myodegenerative phenomena, including two strongly associated with IBM pathogenesis [8]. The most prominent mechanistic hypothesis for IBM myodegeneration implicates abnormal proteostasis, as many proteins associated with neurodegenerative disease (e.g. TDP-43, p62, amyloid-β, and αβ-crystallin) are reported to aggregate in the cytosol of IBM-affected myofibers [2, 12, 23]. Ca^2+^ dysregulation contributes to abnormal proteostasis by promoting mitochondrial reactive oxygen species (ROS) production and perturbing protein folding in the endoplasmic reticulum (ER) lumen [18, 31]. Additionally, activation of Ca^2+^-dependent proteases downstream of Ca^2+^ dysregulation may cause TDP-43 cytosolic mislocalization (a specific finding in IBM vs. other inflammatory myopathies) [57]. Another factor proposed to cause myodegeneration in IBM is mitochondrial dysfunction; accumulation of mtDNA deletions, ultrastructural abnormalities, loss of oxidative phosphorylation enzyme activity, and abnormal mitophagy have been reported in IBM muscle [31, 48]. As with proteostasis, Ca^2+^ dyshomeostasis is closely linked with mitochondrial dysfunction. Excessive Ca^2+^ uptake by the mitochondria can stimulate ROS production, initiate formation of the permeability transition pore, and diminish the inner mitochondrial membrane potential critical for oxidative phosphorylation [7, 13, 31].

The direct link between Ca^2+^ dysregulation, abnormal proteostasis, and mitochondrial dysfunction makes Ca^2+^ regulation a phenomenon of potential pathological significance in IBM. Although Ca^2+^ regulation has not yet been investigated in human cases of IBM, data exist suggesting that some proteins associated with IBM can mediate an insult to Ca^2+^ homeostasis [1, 11, 34, 46]. Additionally, abnormal Ca^2+^ regulation may plausibly stem from membrane pores and osmotic stress, as clonal cytotoxic T-cells (expressing membranolytic enzymes) have been reported in IBM and may have a particularly aggressive phenotype [16, 20, 44]. Given the plausibility of an insult to Ca^2+^ regulation in IBM, we hypothesized that human cases of IBM would display downstream gene and protein-level evidence of alterations consistent with Ca^2+^ dysregulation. In this study, we compared muscle biopsies from IBM patients with those of DM (an inflammatory myopathy without prominent degenerative characteristics) patients and non-myositis controls, investigating a panel of Ca^2+^-regulatory proteins as well as the Ca^2+^ signaling transcriptomic pathway. To explain observed alterations, we investigated proteolytic activation of ubiquitously expressed calpain-1, expression of the skeletal muscle specific calpain-3, and ER stress-induced translational attenuation.

## Materials and methods

### Subjects and tissue samples

All procedures were completed under Institutional Review Board (IRB) approved protocols of the University of Maryland and Johns Hopkins University School of Medicine. Patients from the Johns Hopkins Myositis Center consented to use of their snap-frozen, rectus femoris biopsy samples for IRB-approved research purposes. Patients in the IBM group met ENMC 2011 criteria for clinically defined IBM whereas patients in the DM group met Bohan and Peter criteria [6, 21, 47] (Table 1). Patients in the non-myositis control group (labeled CON in figures) were referred to the Johns Hopkins Myositis Center for suspected myopathy, where they were clinically normal or mild in presentation and had normal biopsies with no pathologic findings suggestive of an inflammatory myopathy.

**Table 1:** Demographic information and serum creatine kinase levels for patients included in this study

	Immunoblots			RNA-sequencing	
	CON (*n* = 5)	DM (*n* = 4)	IBM (*n* = 7)	CON (*n* = 7)	IBM (*n* = 9)
Age at Biopsy (years)					
Mean (SD)	62 (4.2)	64.5 (5.8)	65.6 (7.0)	43 (11.2)	62.3 (11.1)
Ethnicity					
Caucasian (%)	4 (80)	4 (100)	6 (85.7)	6 (85.7)	7 (77.8)
African American (%)	1 (20)	0	1 (14.3)	0	1 (11.1)
Asian (%)	0	0	0	1 (14.2)	1 (11.1)
Sex					
Male (%)	4 (80)	4 (100)	6 (85.7)	3 (42.9)	4 (14.4)
Female (%)	1 (20)	0	1 (14.3)	4 (57.1)	5 (55.6)
Serum Creatine Kinase (U/L)					
Mean (SD)	543 (569)	451 (776)	763 (659)	198 (221.8)	700 (630)

### Analysis of protein expression

Samples were prepared as previously described [10]. Briefly, rectus femoris muscle biopsy samples were transferred from liquid nitrogen directly to a tube containing chilled RIPA buffer (0.15 M NaCl, 0.01 M Tris–HCl pH 8, 0.005 M EDTA, 0.5% Sodium Deoxycholate, 0.1% SDS, 1% Triton-X100) and 1 cOmplete™ protease inhibitor cocktail (Sigma-Aldrich) on ice. Biopsies were minced with sterile lab scissors and further homogenized with a Polytron machine. Homogenates were spun at 4 °C for 10 minutes at 14000 rpm (20000 RCF) and soluble protein extracted. Lysate protein concentration was determined via bicinchoninic acid assay (Thermo Fisher). Samples were prepared to have equal total protein concentration and were aliquoted for storage at −80 °C until analysis. Samples for analysis were solubilized in loading buffer and heated at 100 °C for 5 minutes, and stored at 4 °C until subsequent analyses. Antibodies and concentrations used were: anti-SERCA1 1:1000 (Cell Signaling Technology #12293s), anti-SERCA2 1:1000 (Thermo Fischer #9580s), anti-MCU 1:2000 (Cell Signaling Technology #14997), anti-CSQ 1:2500 (Thermo Fischer PA1-913), anti-RyR1 1:500 (Thermo Fischer MA3-925), anti-DHPR1α 1:2000 (Thermo Fischer MA3-920), anti-STIM1 1:1000 (BosterBio PB9406), anti-LETM1 1:2000 (Santa Cruz sc-271234), anti-Grp78/BiP 1:1000 (BD Transduction 3177p), anti-CHOP 1:1000 (Cell Signaling Technology 2895s), anti-eIF2α 1:2000 (Cell Signaling Technology 9722s), anti-P-eIF2α 1:1000 (Cell Signaling Technology 9721s), anti-calpain-1 1:1000 (Thermo Fischer MA1-12434), anti-calpain-3 (Santa Cruz sc-365277), anti-β-actin 1:1000 (Santa Cruz #47778), and anti-vinculin 1:10000 (Sigma-Aldrich V9131). Antigen-antibody complexes were visualized after incubation with Western Clarity ECL (Bio-Rad) and analyzed using either the Bio-Rad Image Lab™ software or ImageStudioLite™. Equal protein loading was confirmed with total protein staining, as previously discussed [10], as well as with probes for β-actin or vinculin.

### Analysis of mRNA expression and protein to transcript ratios

Samples for RNA sequencing were prepared using a standard TRIzol protocol. Briefly, biopsies were homogenized in TRIzol using 1.4 mm ceramic bead low-binding tubes. After phenol-chloroform extraction, RNA was purified and treated with DNase using the RNeasy Mini Kit (Qiagen). Concentration and quality of the resulting RNA was assessed using standard NanoDrop and TapeStation protocols, respectively. Samples were included in analysis with a RNA quality (RIN^e^) value of 7 or higher. Paired-end libraries were prepared using 50 ng of input RNA with the NeoPrep™ system according to the TruSeq™ Stranded mRNA Library Prep protocol (Illumina) and subsequently analyzed using the Illumina HiSeq 2500 machine. Reads were demultiplexed using Casava 1.8.2 and the quality of the resulting fastq files was tested using FastQC 0.11.2. There was no need to mask or trim the reads, as all nucleotide positions had a median Phred score over 30. Reads were aligned to the reference genome (hg19) using Tophat 2.0.134. The fragments per kilobase of exon per million fragments mapped (FPKM) values of each gene in each group were compared using Cuffdiff (Cufflinks 2.2.1) [52] and the graphical analysis was performed using CummeRbund 2.12.1 [53]. Pathway analysis and upstream regulator analysis was performed with Ingenuity® Pathway Analysis (IPA) software, using standard settings [26]. Post-hoc analysis was performed to compare protein expression relative to mRNA levels; mean protein levels (AU; via densitometry of immunoblot) for the studied Ca^2+^-regulatory proteins were divided by FPKM values for each protein. Group means for IBM and controls were compared using Student’s t test.

### Assessment of calpain-1 autolysis

Calpain-1 autolysis was assessed by quantifying full-length (80 kDa) calpain-1 band densitometry, in arbitrary units, as well as cleaved (78 and 76 kDa) isoforms using a method previously described [36]. Briefly, calpain-1 autolysis is a proxy for enzymatic activity [3], and is defined as the percentage of total calpain-1 in its cleaved isoforms. To facilitate distinction of each individual band, biopsy samples homogenized in the aforementioned RIPA lysis buffer were electrophoresed on lower acrylamide percentage (6-8%) gels over a longer duration (2.5 hours at 120 V).

### Assessment of unfolded protein response activation and translational attenuation

To induce downstream effectors of the UPR, an ER stress sensor, Ire1α, facilitates alternative splicing of X-Box Binding Protein 1 (XBP1) mRNA [40]. We quantified XBP1 splicing and overall XBP1 mRNA expression from RNA-seq data. We also investigated protein expression of Grp78/BiP and C/EBP homologous protein (CHOP), effector molecules of the UPR that are upregulated with ER stress [40]. To quantify translational attenuation signaling, we assessed the validated ratio of phosphorylated eukaryotic initiation factor (eIF2α) to total eIF2α [40].

### Data analysis and statistics

All statistical tests and parameters were established, and all experimental data collection and analysis were performed, while the responsible investigators were blind to sample identity. All protein expression values, as quantified by densitometry, were expressed as fold change vs. controls with error bars representing standard error of the mean (SEM). Student's t test, using pre-specified parameters, was used as the primary moderator of significant differences between experimental groups for immunoblots. Where appropriate, false discovery rate (q-value) was used to adjust the significance of analyses for multiple comparisons [4].

## Results

### Ca^2+^-regulatory protein expression is altered in IBM

**Fig. 1:**
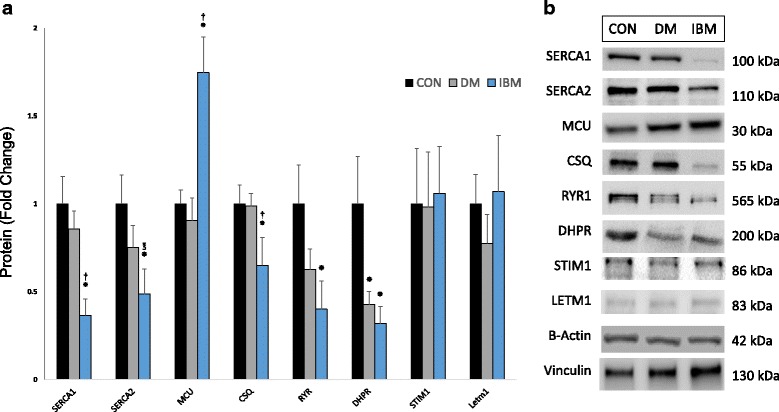
Altered Ca^2+^-regulatory protein expression in IBM. **a** Protein levels of pre-specified panel of proteins, as assessed by immunoblot, expressed as mean + SEM. N = 5, 4, and 7 for non-myositis controls (CON), DM, and IBM, respectively. **b** Representative immunoblots. *P < 0.05 vs CON; ^†^ *P* < 0.05 vs DM; ^℥^ *P* = 0.07 vs DM

We used immunoblotting to investigate the expression of a pre-specified panel of Ca^2+^-regulatory proteins that have been implicated in skeletal myopathy (Fig. 1). The sarco/endoplasmic reticulum Ca^2+^ ATPase (SERCA) proteins, SERCA1 and SERCA2a, are critical intracellular Ca^2+^ buffering proteins in fast and slow skeletal muscle, respectively. The SERCA proteins function to actively transport Ca^2+^ from cytosol to the SR lumen and are subject to cytosolic Ca^2+^ concentration-dependent proteolysis by calpains [49]. Compared with controls, SERCA1 protein was reduced 64% in IBM (*p* < 0.01) and 57% in DM (*p* < 0.01). SERCA2 was reduced 51% in IBM compared to controls (*p* < 0.05) and showed a non-significant trend toward reduction compared to DM (35% decrease, *p* = 0.07). Calsequestrin (CSQ) has an important Ca^2+^-binding function in the SR, serving as a buffer to reduce effective Ca^2+^ concentration in the SR lumen and augment SERCA function [37, 42]. CSQ expression levels were 34% lower in muscles from IBM patients compared to muscles from both controls (*p* < 0.05) and DM (*p* = 0.05). The mitochondrial Ca^2+^ uniporter (MCU) is an inner mitochondrial membrane complex that buffers the cytoplasmic concentration of Ca^2+^ by facilitating entry of Ca^2+^ into the mitochondrial matrix. We detected a 75% increase in MCU expression in IBM vs. both controls (*p* < 0.01) and DM (*p* < 0.01). The skeletal muscle RyR1 is the primary Ca^2+^ release channel of the SR, has altered Ca^2+^ gating after exposure to ROS, and is dynamically regulated by calpain cleavage [15, 45]. We observed 60% lower levels of RyR1 in IBM vs controls (*p* < 0.05), but no difference between IBM and DM (*p* = 0.12). The DHPR is an L-type sarcolemmal Ca^2+^ channel that allows Ca^2+^ influx from the extracellular space and regulates RyR1-dependent Ca^2+^ release during excitation-contraction coupling. DHPR expression was decreased in both IBM (*p* < 0.05) and DM (*p* < 0.05) vs. controls, but did not significantly differ between IBM and DM (*p* = 0.17). We did not detect any differences between groups in expression of leucine zipper and EF-hand containing transmembrane protein (LETM1), a mitochondrial Ca^2+^/H^+^ antiporter, or stromal interaction molecule 1 (STIM1), an SR protein that acts a sensor of Ca^2+^ levels within the SR lumen (all *p* > 0.10). Together, these observed alterations are consistent with elevated basal Ca^2+^ levels in IBM myofibers, which we predicted would also result in transcriptomic alterations.

### Differential Ca^2+^ signaling gene expression and reduced protein per transcript in IBM

**Fig. 2:**
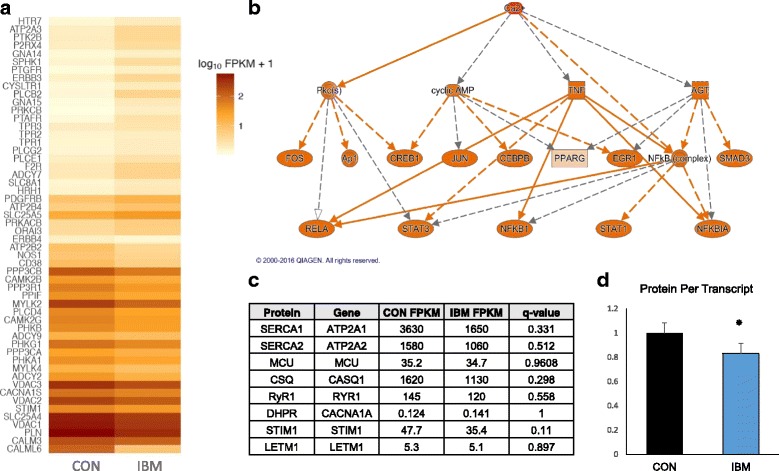
Ca^2+^ signaling transcriptome perturbations in IBM and related post-hoc analyses. **a** Heat map of differentially expressed (q < 0.05) genes in IBM (*n* = 9) versus controls (CON; *n* = 7) from the KEGG Ca^2+^ signaling pathway. **b** Representative network image showing the relationship between Ca^2+^ signaling and transcriptomic regulators; Ca^2+^ abundance was considered a significant (q < 0.01) upstream regulator of observed changes. *Orange nodes* indicate activation consistent with Ca^2+^ abundance. **c** Expression of mRNA (FPKM) for the genes encoding previously immunoblotted Ca^2+^-regulatory proteins, demonstrating no significant (q < 0.05) changes in expression. **d** Reduced protein to transcript ratio amongst Ca^2+^-regulatory proteins in IBM, expressed as mean + SEM. *P < 0.05 versus CON

Paired-end RNA-sequencing analysis of IBM and non-myositis control samples was performed on RNA isolated from muscle biopsies. 183 genes, selected from the KEGG Ca^2+^ signaling pathway (an unbiased gene list), were investigated from whole-transcriptome data. From these 183 genes, 54 (29.5%) were differentially expressed (false discovery rate (q) < 0.05; Fig. 2a). A relevant gene of interest that was not included in the KEGG signaling pathway, PVALB, encodes parvalbumin, a cytosolic Ca^2+^ buffer. PVALB mRNA was increased 2.7-fold in IBM samples (q < 0.001). Dysregulation of the canonical Ca^2+^ signaling pathway, as assessed using Ingenuity Pathway Analysis, was significant (q < 0.01). Using an established statistical approach to relate genes with causal regulatory networks [26], Ca^2+^ abundance was a significant upstream regulator of the observed whole-transcriptome changes (q < 0.01, activation z score = 2.734). Full Ca^2+^ signaling gene list data, with read quantity, fold change, and q values, are available in Additional file 1: Electronic Resource 1. Interestingly, of the six proteins we found to be differentially expressed in IBM vs. controls via immunoblot, none were significantly altered at the mRNA level (all q > 0.10; Fig. 2c). Indeed, when averaging the protein to transcript ratio of the Ca^2+^-regulatory proteins assessed in this study, IBM had significantly less (*p* < 0.05) protein per transcript than control biopsies (Fig. 2d), implicating post-transcriptional down-regulation of these proteins via increased degradation or reduced translation.

### Altered levels of Ca^2+^-activated proteases in IBM

**Fig. 3:**
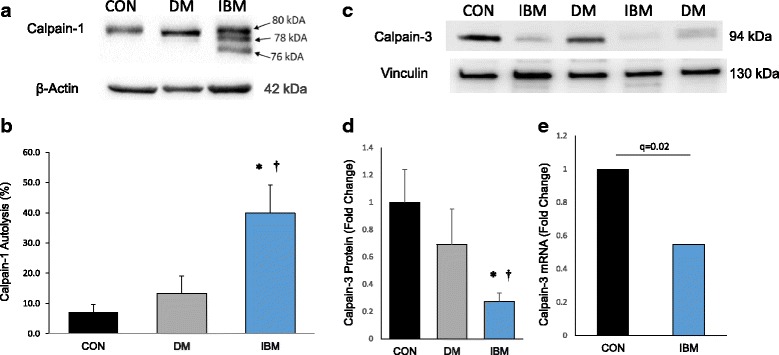
Calpain-1 autolysis and calpain-3 reduction in IBM. **a** Representative immunoblot demonstrating prominent autolysis of 80 kDa native calpain-1 to proteolytically active calpain isoforms. **b** Quantification of 78 and 76 kDa calpain-1 band areas divided by total calpain-1. **c** Representative western blot of native calpain-3 expression. **d** Quantified expression of native calpain-3 protein levels. **e** Transcript expression of CAPN3 gene as determined by RNA-seq. Graphed data expressed as mean + SEM. N of 5, 4, and 7 for non-myositis controls (CON), DM, and IBM, respectively; *P < 0.05 vs CON; ^†^ *P* < 0.05 vs DM

Since our data implicated cytosolic Ca^2+^ elevations in IBM, we hypothesized that Ca^2+^-activated proteolysis may contribute to the decreased protein to transcript ratio amongst Ca^2+^-regulatory proteins. Amongst other functions, the ubiquitously expressed calpain-1 is known to irreversibly cleave SR Ca^2+^ regulatory proteins [45, 49]. Calpain-1 autolyzes at physiologically high (μM) concentrations of Ca^2+^, forming active/proteolytic 78 and 76 kDa isoforms that can be quantified via immunoblot [36, 50]. Total calpain-1 protein expression was not different between groups (*p* > 0.10; data not shown). However, in IBM samples, we detected prominent 78 and 76 kDa bands, reflecting proteolytically active isoforms (Fig. 3a). Chemiluminescent quantification of these cleaved forms, divided by total calpain-1, shows an approximate 4-fold elevation in calpain-1 autolysis in IBM vs. controls (*p* < 0.01) and DM (*p* < 0.05) (Fig. 3b). Unlike calpain-1, the skeletal muscle specific calpain-3 does not cleave Ca^2+^-regulatory proteins. Alternatively, calpain-3 appears to broadly support cellular Ca^2+^ homeostasis by preventing degradation of SERCA proteins, supporting RyR1 function, and stabilizing the sarcomeric triad [17, 39, 51]. Of interest, loss-of-function mutations in calpain-3 are known to cause limb girdle muscular dystrophy type 2a (LGMD2a; also called calpainopathy) [27], and transgenic reduction of calpain-3 in mice causes severe Ca^2+^ dysregulation [54]. At the mRNA level, expression of calpain-3 was ~50% lower in IBM samples vs. controls (q < 0.05). At the protein level, native calpain-3 was reduced 66% in IBM vs. controls (*p* < 0.01) and 50% vs. DM (*p* < 0.05). DM and controls did not significantly differ in calpain-3 protein expression (*p* = 0.22).

### Translational attenuation downstream of the unfolded protein response in IBM

**Fig. 4:**
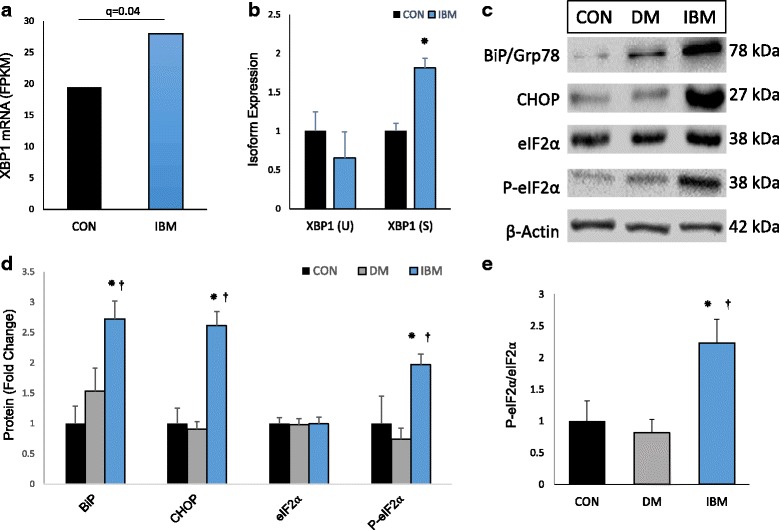
Activation of the UPR and downstream translational attenuation in IBM. **a** XBP1 transcript levels (RNA-seq). **b** Isoform-specific expression of ubiquitous (U) and spliced (S) XBP1 transcript (RNA-seq). **c** Representative immunoblots of UPR effector molecules **d** Quantification of UPR effector protein levels. **e** Relative phosphorylation of eIF2α, a marker of translational attenuation. Graphed data expressed as mean + SEM. N of 5, 4, and 7 for non-myositis controls (CON), DM, and IBM, respectively; *P < 0.05 vs CON; ^**†**^ *P* < 0.05 vs DM

In addition to proteolysis, we hypothesized that altered translation might play a role in the observed decrease in protein to transcript ratio for Ca^2+^-regulatory proteins. Protein accumulation and Ca^2+^ dysregulation activate ER stress signaling and the UPR to restore normal proteostasis. Initiated by alternative splicing of XBP1, the UPR up-regulates effector molecules (e.g. chaperone proteins) but broadly halts protein translation through phosphorylation of eIF2α. Compared with controls, XBP1 mRNA was upregulated and preferentially spliced to its UPR effector form in IBM (*p* < 0.05; Fig. 4a, b). IBM-specific over-expression of two UPR effectors, Grp78/BiP (a heat-shock protein) and CHOP (a downstream pro-apoptotic transcription factor) further supported the evidence for UPR activation at the protein level (*p* < 0.01; Fig. 4c, d). Total eIF2α expression was not different between groups, but relative phosphorylation of eIF2α was ~2.5-fold elevated in IBM samples compared with controls (*p* < 0.05) and DM (*p* < 0.01) (Fig. 4d, e). These data suggest that the UPR is activated in IBM, leading to reduced protein translation.

## Discussion

Ca^2+^ is a ubiquitous second messenger in skeletal muscle, regulating processes as varied as secretion, contraction, and apoptosis. In healthy myofibers, intracellular Ca^2+^ concentration is maintained in a tight homeostasis by various Ca^2+^-regulatory proteins which function as ion sensors, channels, transporters, and buffers. Diminished Ca^2+^ regulation, typically resulting in pathologically high levels of cytosolic Ca^2+^, plays a pathogenic role in several neurodegenerative and neuromuscular diseases and has many upstream causes [54, 56]. For example, pathogenic Ca^2+^ dysregulation is linked with RyR1 alterations in Alzheimer’s disease [15], mutant protein-induced mitochondrial dysfunction in Huntington’s disease [41], and sarcolemma fragility in dystrophinopathies [54]. Pathologic downstream effects of prolonged Ca^2+^ dysregulation include mitochondrial dysfunction and abnormal proteostasis, two likely contributors to myodegeneration in IBM [31]. In this investigation, we report alterations in several proteins and genes associated with Ca^2+^ regulation in IBM muscle, associate these changes with Ca^2+^ overabundance, and identify potential mechanisms contributing to Ca^2+^ dysregulation. This novel study of Ca^2+^ regulation in myositis patients strongly supports the hypothesis that Ca^2+^ dysregulation is present in and pathologically relevant to IBM.

Compared with control and DM muscle biopsies, IBM samples had decreased SERCA1, SERCA2, and CSQ protein and increased MCU protein, supporting mechanisms by which both cytosolic and mitochondrial Ca^2+^ concentrations are likely elevated. Compared with controls, both DM and IBM had reduced levels of RyR1 and DHPR protein, which may reflect a non-specific change associated with muscle damage or inflammation. Of note, reduced protein levels of SERCA1, the fast-twitch SERCA isoform, was previously reported in a proteomic study of IBM that describes broad expression reductions in fast twitch proteins [43]. Interestingly, in this study, the 30 most reduced proteins in IBM (by fold-change) included SERCA1 (#11, 0.53-fold) and CSQ (#15, 0.58-fold) [43]. We observed similar decreases in SERCA1 and CSQ; however, the reduction in SERCA expression in our IBM patients did not predominantly affect the fast isoform, as the SERCA1 to SERCA2 ratio was unchanged between groups (Additional file 2: Electronic Resource 2). Altered expression of Ca^2+^-regulatory proteins have not been reported in the three other IBM proteomic studies to date, although these studies each report alterations in only 29 proteins or fewer [24, 29, 30], likely reflecting limitations of the 2-D gel electrophoresis approach to detect differential protein expression in muscle biopsies.

Prior studies have investigated the IBM transcriptome, although no study to our knowledge has reported on alterations in the Ca^2+^ pathway. Primarily, these studies use microarrays to screen for genes with high comparative expression changes vs. controls and other inflammatory myopathies. Our approach differed, pre-specifying an unbiased list of genes (the KEGG Ca^2+^ signaling pathway) from which to extract transcript expression information and perform bioinformatics analysis. This approach allowed for detection of subtle but still highly significant expression changes at the gene and pathway level. Our analyses revealed the Ca^2+^ signaling canonical pathway to be significantly altered in IBM, with 54 of 183 genes (29.5%) differentially expressed vs. control muscle after correction for multiple comparisons. Several gene expression alterations imply myocellular compensation for the loss of Ca^2+^ homeostasis, such as upregulation of PVALB (a Ca^2+^ buffer) and suppression of phospholamban (PLN; a SERCA-inhibitory peptide), which would increase cytosolic Ca^2+^ buffering and SR Ca^2+^ uptake, respectively. Using the upstream regulator analysis function within IPA, which predicts activation status of upstream molecules using a large dataset of causal experiment data, whole-transcriptome changes observed in IBM were determined to be highly consistent with myocellular Ca^2+^ abundance [26].

Perhaps our most interesting finding is that IBM displays alterations in the activation status and expression of Ca^2+^-activated proteases. Robust (~4-fold) elevations in autolytic activation of the Ca^2+^-activated protease calpain-1 in IBM provides strong evidence of abnormally high cytosolic Ca^2+^ concentration, and may explain specific protein expression decreases (e.g. SERCA1, SERCA2, and RyR1) that were out of proportion with their respective mRNA decreases. It is worth noting again that neither calpain-1 transcript nor overall protein level were increased in IBM, indicating that calpain-1 abnormalities in IBM are primarily post-translational. In neurons, moderate calpain-1 activation (as we observe in IBM samples) causes cleavage of TDP-43 into aggregation-prone fragments, promoting the TDP-43 cytoplasmic mislocalization (TDP-43 proteinopathy) observed in amyotrophic lateral sclerosis [1, 57]. As TDP-43 proteinopathy causes pathology in human cells by altering RNA dynamics [25, 33] and is a specific histology finding in IBM muscle vs. other inflammatory myopathies [23], calpain-mediated TDP-43 cleavage may reflect a highly novel upstream pathogenic mechanism in IBM. However, no experiments have yet confirmed that this relationship between calpain and TDP-43 is conserved in skeletal muscle. The other Ca^2+^-activated protease investigated in this study, calpain-3, was reduced in IBM samples vs. controls and DM. This finding is consistent with a hypothesis proposed in a previous proteomic study, in which calpain-3 substrates were among the few over-expressed proteins in IBM samples [43]. Contrary to calpain-1, which functions primarily through proteolysis, calpain-3 has several important non-proteolytic functions, including a prominent role in sarcomere remodeling [39]. Moreover, calpain-3 was recently reported to associate with and prevent proteasomal degradation of SERCA proteins [51]. Thus, diminished calpain-3 expression provides a novel mechanism by which Ca^2+^ dysregulation may be initiated or exacerbated and may have broader implications for myofiber adaptation to damage [28, 39].

Abnormal proteostasis and downstream activation of the UPR have long been hypothesized to contribute to IBM pathology [55]. Supporting this theory, IBM biopsies have been reported to display activation of the UPR by XBP1 splicing and elevated expression of several ER stress-induced molecules [38, 55]. As previously discussed, the UPR suppresses translation through phosphorylation of eIF2α, but this had not been investigated in IBM muscle. Consistent with previous studies, our IBM patients displayed evidence for UPR activation, including XBP1 induction, XBP1 mRNA splicing, and upregulation of the ER stress-inducible proteins BiP/GRP78 and CHOP. As hypothesized, we also observed increased eIF2α phosphorylation in IBM samples vs. controls and DM, indicating that the UPR is suppressing translation in these samples via inhibition of the eIF2α translation initiation factor. In the context of increased calpain-1 proteolysis and diminished expression of calpain-3, translational attenuation in IBM muscle likely potentiates reductions in contractile and Ca^2+^-regulatory proteins. Of additional interest, activation of the UPR can induce local inflammation, which may contribute to repression of the gene encoding calpain-3 (CAPN3), further linking these phenomena [19].

**Fig. 5:**
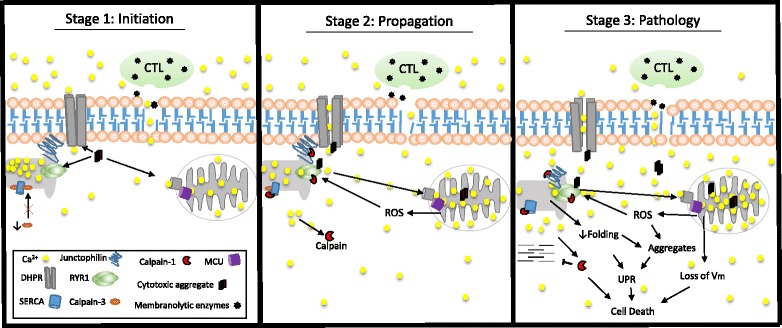
A theoretical mechanism of progressive Ca^2+^ dysregulation in IBM and downstream pathological relevance. **Stage 1:** An initial insult to Ca^2+^ regulation may come from immune-mediated membrane injury (pictured: cytotoxic T-lymphocyte), permeabilization of Ca^2+^ channels by abnormal proteins, and/or reduction in calpain-3. **Stage 2:** Ca^2+^ elevations in the cytosol are then propagated through ROS production, SR Ca^2+^ leak, and calpain proteolysis of Ca^2+^-regulatory proteins. **Stage 3:** Prolonged Ca^2+^ dyshomeostasis causes and/or exacerbates the inter-related phenomena of mitochondrial dysfunction, UPR signaling, protein aggregate formation, and contractile dysfunction

The relationship between Ca^2+^ dysregulation and other pathogenic mechanisms in IBM is complex, but possibly significant for disease progression. To illustrate this relationship, we propose a theoretical three-phase mechanism of Ca^2+^ dysregulation in IBM that integrates our data with the literature (Fig. 5). While this model does not attempt to explain what causes an individual to develop IBM, it does connect many pathological elements observed in IBM biopsies. In the initiation [of Ca^2+^ dysregulation] phase, one or more of the following may provide an initial insult to myofiber Ca^2+^ homeostasis: i) sarcolemma damage and subsequent osmotic stress stemming from expanded and potentially-aggressive cytotoxic T-lymphocytes (CTLs) [16, 20, 44]; ii) alterations in Ca^2+^ channel function and/or mitochondrial Ca^2+^ buffering due to cytotoxic protein oligomers or fibrils [1, 11, 34, 46]; and/or iii) diminishment of SERCA function caused by calpain-3 reduction [39, 51]. Without amelioration of such insult(s) to Ca^2+^ homeostasis, Ca^2+^ dysregulation progresses in the propagation phase. Mitochondria, susceptible to injury in IBM due to conditions of inflammation, mtDNA abnormalities, and impaired proteostasis, are damaged by excessive Ca^2+^ influx [7, 31, 32, 48]. This stimulates ROS production, promoting further Ca^2+^ efflux from the SR [15] and ER stress signaling [32]. Cytosolic Ca^2+^ elevations potentiate the activation of calpains (e.g. calpain-1), which cleave Ca^2+^-regulatory proteins like SERCA1 [49, 50]. Ca^2+^ and redox imbalances in the ER perturb protein folding, inducing UPR signaling [18, 32]. Together, impairment of mitochondrial buffering, degradation of Ca^2+^-regulatory proteins, and suppression of translation cause progressive increases in cytosolic Ca^2+^. In the myofiber pathology phase, mitochondrial damage has diminished the capacity for oxidative phosphorylation through reduction of the inner mitochondrial membrane potential, prolonged ER stress has altered myofiber viability through induction of pro-apoptotic factors (e.g. CHOP), and calpain-1 activation has directly contributed to weakness by degrading proteins involved in excitation-contraction coupling (e.g. RyR1).

This study provides new insights into a potential contributor to IBM pathogenesis, but further research is needed. Future studies may investigate the initiation of Ca^2+^ dysregulation in IBM by interrogating the association between indicators of Ca^2+^ dysregulation (e.g. calpain-1 activation) and markers of potential upstream causes (e.g. protein aggregation) and/or attempting to functionally recapitulate aspects of this phenomenon in model systems. A limitation of this study was the use of a restricted number of biopsies, from different patients, in immunoblot and RNA-seq studies. This limits the strength of protein to transcript comparisons, although our finding of reduced protein to transcript amongst Ca^2+^-regulatory proteins was well supported by subsequent experiments (i.e. calpain-1 activation and eIF2α phosphorylation). Finally, while we did not find substantial Ca^2+^-associated abnormalities in our myositis control (DM) patients, future studies may look to address whether the pathologies of other inflammatory myopathy subsets (e.g. patients with necrotizing myopathies or specific autoantibodies) include Ca^2+^ dysregulation.

## Conclusion

This investigation provides data, from whole-transcriptome analysis to specific proteins alterations, that implicate Ca^2+^ dysregulation in the myocellular pathology of sporadic IBM. While it is still unclear which theoretical insult(s) are upstream of Ca^2+^ dysregulation in IBM, our data suggest that this phenomenon is propagated by reduced expression of calpain-3, abnormal proteolysis secondary to calpain-1 activation, and decreased protein translation downstream of the UPR. While Ca^2+^ dysregulation is unlikely to be a primary pathogenic mechanism in IBM, it may contribute to muscle atrophy and weakness through its pleiotropic effects on protease dynamics, gene expression, myocellular proteostasis, and mitochondrial function. As such, future investigations may investigate if targeted treatment aimed to restore Ca^2+^ homeostasis and/or limit the downstream effects of prolonged Ca^2+^ dysregulation may be a viable therapeutic strategy in IBM.

## Additional files

Additional file 1: Electronic Resource 1: RNA-sequencing data for genes in the KEGG Ca^2+^ signaling pathway, including gene name and locus, mRNA expression (Fragments Per Kilobase of transcript per Million mapped reads), fold change, and comparison false discovery rate (q-value). (PDF 289 kb)

Additional file 2: Electronic Resource 2: The ratio of SERCA1 to SERCA2 protein is unaltered between groups (all *P* > 0.10), suggesting a lack of fiber-type specificity in the reduction of SERCA proteins. (DOC 50 kb)
