# Supplementary material for: Calcium dysregulation, functional calpainopathy, and endoplasmic reticulum stress in sporadic inclusion body myositis
Source: Acta Neuropathol Commun. 2017 Mar 22;5:24. doi: 10.1186/s40478-017-0427-7 (PMC5363023; doi:10.1186/s40478-017-0427-7)
Supplement: Supplementary file 1 — Electronic Resource 1: RNA-sequencing data for genes in the KEGG Ca2+ signaling pathway, including gene name and locus, mRNA expression (Fragments Per Kilobase of transcript per Million mapped reads), fold change, and comparison false discovery rate (q-value). (PDF 289 kb) [file 40478_2017_427_MOESM1_ESM.pdf]

Gene	Locus	CON (FPKM)	IBM (FPKM)	Log_2 Fold Change	q-value
ADCY1	chr7:45613738-45762714	1.73	1.08	-0.68	0.067567
ADCY2	chr5:7396342-7830194	32.56	16.27	-1	0.000546
ADCY3	chr2:25013135-25142055	2.45	4.23	0.79	0.072389
ADCY4	chr14:24787554-24804277	4.48	4.97	0.15	0.685466
ADCY7	chr16:50300450-50352045	1.47	3.93	1.42	0.000546
ADCY8	chr8:131792546-132052835	0.04	0.03	-0.44	1
ADCY9	chr16:4012649-4166186	14.86	10.36	-0.52	0.028971
ADORA2A	chr22:24666784-24890783	0.71	1.9	1.42	0.322656
ADORA2B	chr17:15848230-15879210	0.3	0.55	0.86	1
ADRA1A	chr8:26605666-26722922	0.15	0.24	0.66	1
ADRA1B	chr5:159343739-159400017	0.5	0.69	0.48	1
ADRA1D	chr20:4201277-4229659	0.19	0.27	0.53	1
ADRB1	chr10:115803805-115806667	0.13	0.36	1.49	1
ADRB2	chr5:148206155-148208197	7.07	6.32	-0.16	0.655247
ADRB3	chr8:37820513-37824184	0	0.01	inf	1
AGTR1	chr3:148415657-148460790	6.91	5.47	-0.34	0.293855
ATP2A1	chr16:28889808-28936532	3326.51	1458.46	-1.19	0.465148
ATP2A2	chr12:110719031-110788897	1535.88	986.17	-0.64	0.320364
ATP2A3	chr17:3827162-3867758	1	3.44	1.79	0.000546
ATP2B1	chr12:89981825-90049844	5.69	6.15	0.11	0.737056
ATP2B2	chr3:10365706-10547268	8.39	3.27	-1.36	0.000546
ATP2B3	chrX:152801579-152848387	0	0.01	inf	1
ATP2B4	chr1:203595914-203713209	5.04	7.92	0.65	0.006254
AVPR1A	chr12:63536538-63546590	0.8	1.08	0.43	0.224343
AVPR1B	chr1:206224282-206231482	0	0	0	1
BDKRB1	chr14:96722546-96731100	0.05	0.41	3.05	1
BDKRB2	chr14:96671134-96710666	0.3	0.73	1.27	1
CACNA1A	chr19:13317255-13617274	0.12	0.13	0.12	1
CACNA1B	chr9:140762376-141019076	0	0.01	1.38	1
CACNA1C	chr12:2162415-2807115	0.26	0.3	0.2	1
CACNA1D	chr3:53529075-53846492	0.02	0.03	0.88	1
CACNA1E	chr1:181452685-181775921	0.01	0.11	3.97	1
CACNA1F	chrX:49061522-49089833	0.01	0.04	1.96	1
CACNA1G	chr17:48633567-48704832	0.03	0.02	-0.61	1
CACNA1H	chr16:1203240-1275254	0.74	1.03	0.47	0.248026
CACNA1I	chr22:39966757-40085740	0	0.01	inf	1
CACNA1S	chr1:201008639-201081694	79.77	50.67	-0.65	0.01641
CALM1	chr14:90863326-90874619	202.49	141.87	-0.51	0.095037
CALM2	chr2:47387220-47404075	176.75	158.98	-0.15	0.628781
CALM3	chr19:47104511-47114039	163.21	112.72	-0.53	0.032458
CALML3	chr10:5556206-5568231	0	0.17	inf	1
CALML5	chr10:5540657-5541533	0	0	0	1
CALML6	chr1:1846265-1848733	104.73	7.53	-3.8	0.000546
CAMK2A	chr5:149599053-149669403	47.99	38.99	-0.3	0.291482
CAMK2B	chr7:44256748-44365230	32.57	18.74	-0.8	0.001009

CAMK2D	chr4:114372187-114683083	22.48	27.84	0.31	0.299033
CAMK2G	chr10:75572258-75634349	53.88	28.23	-0.93	0.000546
CAMK4	chr5:110559946-110820748	0.06	0.29	2.21	1
CASQ1	chr1:160160284-160171676	1529.32	1037.74	-0.56	0.205607
CASQ2	chr1:116242625-116311426	110.08	125.33	0.19	0.552794
CCKAR	chr4:26483017-26492042	0	0.01	inf	1
CCKBR	chr11:6280903-6293357	0.03	0	-2.74	1
CD38	chr4:15779930-15850706	9.36	4.65	-1.01	0.00217
CHRM1	chr11:62676150-62689012	0	0	-0.29	1
CHRM2	chr7:136553398-136849088	0	0	inf	1
CHRM3	chr1:239792372-240072717	0.07	0.07	0.13	1
CHRM5	chr15:34158427-34357287	0	0.01	1.39	1
CHRNA7	chr15:32322685-32462384	0.13	0.05	-1.33	1
CYSLTR1	chrX:77526968-77583188	0.37	1.18	1.69	0.000546
CYSLTR2	chr13:49227846-49283498	0.02	0.21	3.33	1
DRD1	chr5:174867674-174871163	0.03	0.04	0.58	1
DRD5	chr4:9783257-9785633	0	0	inf	1
EDNRA	chr4:148402068-148466106	1.95	2.91	0.57	0.106577
EDNRB	chr13:78393071-78549664	1.82	1.8	-0.01	0.981696
EGFR	chr7:55086724-55275031	1.48	1.44	-0.04	0.943468
ERBB2	chr17:37844336-37884915	8.88	9.53	0.1	0.760046
ERBB3	chr12:56473808-56497291	0.75	3.43	2.19	0.000546
ERBB4	chr2:212240441-213403352	0.92	0.58	-0.67	0.043735
F2R	chr5:76011867-76031595	1.46	3.89	1.42	0.000546
GNA11	chr19:3094407-3124000	26.89	20.81	-0.37	0.133576
GNA14	chr9:80037994-80263232	0.4	0.93	1.21	0.024384
GNA15	chr19:3136029-3163767	0.22	1.35	2.61	0.027851
GNAL	chr18:11689013-11908796	3.6	2.62	-0.46	0.659745
GNAQ	chr9:80331189-80646365	4.79	6.1	0.35	0.186374
GNAS	chr20:57393972-57486251	982.09	683.35	-0.52	0.238112
GRIN1	chr9:140033608-140064491	0	0.01	inf	1
GRIN2A	chr16:9847261-10276611	0.02	0.02	-0.35	1
GRIN2C	chr17:72838161-72856966	0.2	0.16	-0.29	1
GRIN2D	chr19:48898131-48948188	0.04	0.14	1.85	1
GRM1	chr6:146348917-146758734	0.01	0.01	-0.26	1
GRM5	chr11:88237743-88796846	0	0.03	inf	1
GRPR	chrX:16141423-16171641	0.03	0.08	1.31	1
HRH1	chr3:11178778-11304939	0.66	1.47	1.15	0.006254
HRH2	chr5:175085039-175113245	0.07	0.17	1.16	1
HTR2A	chr13:47405676-47471211	0.18	0.1	-0.89	1
HTR2B	chr2:231921577-232037540	0.12	0.24	0.94	1
HTR2C	chrX:113818550-114144624	0	0.01	inf	1
HTR4	chr5:147830594-148034090	0.04	0.04	0.22	1
HTR5A	chr7:154858778-154879102	0	0	inf	1
HTR6	chr1:19991779-20007459	0.01	0.01	-1.35	1
HTR7	chr10:92500575-92617671	0.45	1.23	1.44	0.037357
ITPKA	chr15:41786055-41795757	0.22	0.59	1.41	1

ITPKB	chr1:226819390-226926876	3.46	4.54	0.39	0.161377
ITPKC	chr19:41223007-41255828	2.04	2.32	0.18	0.78157
ITPR1	chr3:4535031-4889524	0.58	1.03	0.84	0.010509
ITPR2	chr12:26488269-26986131	0.63	1	0.66	0.025887
ITPR3	chr6:33589155-33664348	1.03	1.76	0.77	0.005359
LHCGR	chr2:48757063-49003656	0.04	0.03	-0.17	1
LTB4R2	chr14:24769059-24787242	0.38	1.06	1.48	0.336845
MCU	chr10:74451888-74647452	34.09	33.92	-0.01	0.984781
MICU1	chr10:74127083-74385949	36.06	33.73	-0.1	0.77688
MYLK	chr3:123304359-123603149	6.34	7.14	0.17	0.70161
MYLK2	chr20:30407177-30422500	234.95	100.91	-1.22	0.000546
MYLK3	chr16:46736193-46797158	11.3	8.89	-0.35	0.21382
MYLK4	chr6:2663862-2751154	15.04	8.95	-0.75	0.010042
NOS1	chr12:117645946-117799607	6.8	3.82	-0.83	0.001009
NOS2	chr17:26083791-26127555	0.11	0.12	0.17	1
NOS3	chr7:150688143-150721586	1.14	1.44	0.34	0.465483
NTSR1	chr20:61340188-61394123	0.01	0.02	0.32	1
ORAI1	chr12:122064454-122079946	44.17	33.37	-0.4	0.10797
ORAI2	chr7:102073976-102105321	0.27	0.62	1.22	1
ORAI3	chr16:30960404-30966259	2.88	4.66	0.69	0.037211
OXTR	chr3:8792094-8811300	0.07	0.16	1.13	1
P2RX1	chr17:3799884-3819960	0.17	0.42	1.35	1
P2RX2	chr12:133195365-133198972	0	0.01	inf	1
P2RX3	chr11:57105840-57137549	0.09	0.06	-0.61	1
P2RX4	chr12:121647663-121671909	0.99	2.73	1.46	0.001009
P2RX5	chr17:3539761-3599698	5.15	6	0.22	0.833018
P2RX6	chr22:21369441-21382302	13.3	11.4	-0.22	0.44409
P2RX7	chr12:121570621-121624354	0.12	0.73	2.61	1
PDE1A	chr2:183004761-183387572	1.65	1.7	0.04	0.94269
PDE1B	chr12:54943176-54973023	0.58	0.95	0.71	0.141063
PDE1C	chr7:31790792-32339016	0.13	0.23	0.82	1
PDGFRA	chr4:55095263-55164412	5.61	7.06	0.33	0.199761
PDGFRB	chr5:149493401-149535422	7.75	13.72	0.82	0.000546
PHKA1	chrX:71798663-71934029	38.6	23.81	-0.7	0.007821
PHKA2	chrX:18908413-19002480	2.87	2.09	-0.46	0.121466
PHKB	chr16:47495209-47735434	44.35	27.6	-0.68	0.007821
PHKG1	chr7:56131916-56160689	79.25	46.13	-0.78	0.007821
PHKG2	chr16:30759619-30787628	2.26	2.59	0.2	0.832272
PLCB1	chr20:8112911-8865547	2.1	1.47	-0.52	0.080177
PLCB2	chr15:40580097-40600174	0.48	3.87	3.01	0.000546
PLCB3	chr11:64018994-64036924	1.47	2.01	0.45	0.177128
PLCB4	chr20:9049700-9461462	1.17	0.98	-0.25	0.555882
PLCD1	chr3:38048986-38071154	1.34	2.12	0.66	0.107776
PLCD3	chr17:43189007-43209900	39.69	30.85	-0.36	0.158127
PLCD4	chr2:219472487-219501909	46.55	24.68	-0.92	0.000546
PLCE1	chr10:95753745-96088148	0.9	2.02	1.17	0.000546
PLCG1	chr20:39657461-39804357	4.37	5.88	0.43	0.245971

PLCG2	chr16:81812862-81996298	0.55	1.1	1	0.004468
PLCZ1	chr12:18836109-18890993	0	0	0	1
PLN	chr6:118781934-119031238	586.48	346.8	-0.76	0.013907
PPIF	chr10:81107219-81115089	37.56	21.29	-0.82	0.000546
PPP3CA	chr4:101944586-102268628	24.77	16.08	-0.62	0.007046
PPP3CB	chr10:75196185-75335433	141.63	66.51	-1.09	0.001806
PPP3CC	chr8:22298482-22398657	29.38	21.26	-0.47	0.06167
PPP3R1	chr2:68405988-68479651	61.42	32.61	-0.91	0.000546
PPP3R2	chr9:104331633-104500862	0.01	0	-0.81	1
PRKACA	chr19:14202506-14228559	140.88	106.71	-0.4	0.157087
PRKACB	chr1:84543657-84704181	2.92	5.12	0.81	0.003549
PRKACG	chr9:71627448-71629039	0	0	0	1
PRKCA	chr17:64298925-64806862	2	1.84	-0.12	0.75853
PRKCB	chr16:23847299-24231932	0.16	0.99	2.63	0.000546
PRKCG	chr19:54385466-54410901	0	0.02	inf	1
PTAFR	chr1:28473676-28520447	0.35	1.92	2.45	0.000546
PTGER1	chr19:14583277-14586174	0.08	0.12	0.53	1
PTGER3	chr1:71318035-71546972	0.29	0.91	1.67	0.16676
PTGFR	chr1:78956727-79006386	0.26	0.88	1.76	0.000546
PTK2B	chr8:27168998-27316908	1.08	3.45	1.67	0.000546
RYR1	chr19:38924339-39078204	136.08	98.07	-0.47	0.29882
RYR2	chr1:237205701-237997288	0.03	0.05	0.88	1
RYR3	chr15:33603162-34158304	1.67	1.92	0.2	0.588194
SLC25A31	chr4:128651554-128695447	0.03	0.01	-2.16	1
SLC25A4	chr4:186064416-186071538	422.37	244.23	-0.79	0.027851
SLC25A5	chrX:118599995-118605359	18.92	30.86	0.71	0.006512
SLC25A6	chrY:1455044-1461039	0	0	0	1
SLC8A1	chr2:40144773-40739575	0.58	1.24	1.11	0.004468
SLC8A2	chr19:47931278-47975307	0.02	0.11	2.51	1
SLC8A3	chr14:70510933-70655787	8.35	6.69	-0.32	0.308667
SPHK1	chr17:74380689-74383941	0.6	2.72	2.17	0.000546
SPHK2	chr19:49118583-49133663	1.66	2.7	0.7	0.492853
STIM1	chr11:3876932-4114440	45.36	32.04	-0.5	0.049575
STIM2	chr4:26862312-27027003	2.57	3.42	0.41	0.157997
TACR1	chr2:75273589-75426645	0.12	0.35	1.59	1
TACR2	chr10:71163957-71176674	0.17	0.35	0.99	1
TACR3	chr4:104510624-104640973	0.01	0.02	1.06	1
TBXA2R	chr19:3594503-3606831	1.1	1.05	-0.06	0.931801
TNNC1	chr3:52485106-52488057	9892.45	6816.18	-0.54	0.368941
TNNC2	chr20:44451854-44455953	11314	5482.57	-1.05	0.057154
TRHR	chr8:110099652-110131812	0	0.01	inf	1
VDAC1	chr5:133307565-133340824	480.89	271.18	-0.83	0.005359
VDAC2	chr10:76969911-76991207	220.77	130	-0.76	0.001806
VDAC3	chr8:42249278-42263455	303.48	166.29	-0.87	0.000546

From article in Acta Neuropathologica: Calcium dysregulation, functional calpainopathy, and endoplasmic reticulum stress in sporadic Inclusion Body Myositis. David R. Amici[1,2], Iago Pinal-Fernandez[2,3], Davi A. G. Mázala[1], Thomas E. Lloyd[2,3], Andrea M. Corse[3], Lisa Christopher-Stine[3], Andrew L. Mammen[2,3]*, Eva R. Chin[1]*

[1]Dept. of Kinesiology, University of Maryland College Park. College Park, MD, USA.
[2]Muscle Disease Unit, National Institute of Arthritis and Musculoskeletal and Skin Diseases. Bethesda, MD, USA.
[3]Department of Neurology, Johns Hopkins University School of Medicine. Baltimore, MD, USA.

Correspondence to Eva R Chin (erchin@umd.edu)
