# Supplementary material for: Calcium dysregulation, functional calpainopathy, and endoplasmic reticulum stress in sporadic inclusion body myositis
Source: Acta Neuropathol Commun. 2017 Mar 22;5:24. doi: 10.1186/s40478-017-0427-7 (PMC5363023; doi:10.1186/s40478-017-0427-7)

Electronic resource 2

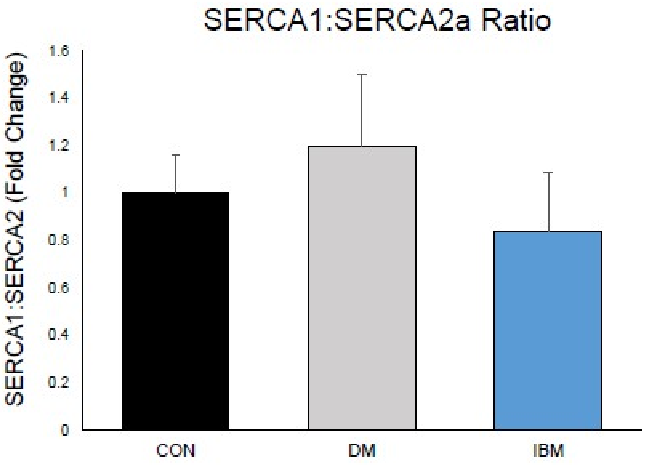

Supplement: Supplementary file 2 [file 40478_2017_427_MOESM2_ESM.doc]
